# Supplementary material for: Heterogeneity in proline hydroxylation of fibrillar collagens observed by mass spectrometry
Source: PLoS One. 2021 Aug 31;16(8):e0250544. doi: 10.1371/journal.pone.0250544 (PMC8407550; doi:10.1371/journal.pone.0250544)
Supplement: S1 File — (DOCX) [file pone.0250544.s001.docx]

**S1 File: The complete sequencing results**

In the following tables, P_y_ is unhydroxylated Pro in a Y-position, O_x_ is hydroxylated proline in an X-position, and O_y_ is the (expected) hydroxylated proline in a Y-position. In cases where the residue having the +16 mass cannot be assigned unambiguously, the P_y_ and/or O_x_ residue, as well as the ion fragments containing the residue are marked by italic. The numbering of the residues starts from the first Gly of the triple helix domain (Gly-X-Y repeats); the positions of residues of the N-telopeptide are labeled as negative numbers relative to the first Gly.

**S1 Table: α1(I) chain of rat tail tendon, commercial sample, first trial (RC1A1_a)**

| **m/z ^+1^** | **Score** | **P_y_** | **O_x_** | **O_y_** | **Peptide** |
| --- | --- | --- | --- | --- | --- |
| 783.4359 | 43 |  |  | 561 | ^556^GAAGLOGPK |
| 836.4373 | 57 |  |  |  | ^907^GPAGPQGPR |
| 886.4377 | 62 |  |  |  | ^184^GSEGPQGVR |
| 1088.5371 | 64 |  |  | 318 | ^316^GFOGADGVAGPK |
| 1161.5721 | 67 |  |  | 405 | ^397^GQAGVMGFOGPK |
| 1258.5910 | 61 |  |  | 366,369 | ^361^GLTGSOGSOGPDGK |
| 1306.6386 | 92 |  |  | 249 | ^238^GPSGPQGPSGAOGPK |
| 1322.6335 | 65 |  | 245 | 249 | ^238^GPSGPQGOSGAOGPK |
| 1328.6481 | 74 | 804 |  | 795,798 | ^793^GFOGLOGPSGEPGK |
| 1443.6975 | 126 |  |  | 693,699 | ^688^GAAGPOGATGFOGAAGR |
| 1451.6761 | 63 |  |  | 297,303,306 | ^295^GEOGPSGLOGPOGER |
| 1532.7816 | 92 |  |  |  | ^889^GETGPAGPAGPIGPAGAR |
| 1680.7572 | 87 |  |  | 444 | ^435^DGEAGAQGAOGPAGPAGER |
| 1832.8596 | 51 |  |  | 819,825,831 | ^817^GPOGPMGPOGLAGPOGESGR |
| 1840.9188 | 50 |  |  | 708,717,720 | ^705^VGPOGPSGNAGPOGPOGPVGK |
| 1856.9137 | 61 |  | 707 | 708,717,720 | ^705^VGOOGPSGNAGPOGPOGPVGK |
| 2136.9905 | 81 |  |  | 759,768,771 | ^757^GSOGADGPAGSOGTOGPQGIAGQR |
| 2185.0520 | 74 |  |  | 867,870,873,876 | ^859^GETGPAGPOGAOGAOGAOGPVGPAGK |
| 2228.9837 | 54 |  |  | 537,540,546,552 | ^532^GDTGAOGAOGSQGAOGLQGMOGER |
| 2244.9786 | 67 |  |  | 537,540,546,552 | ^532^GDTGAOGAOGSQGAOGLQGMOGER |
| 2307.1364 | 58 |  |  | 663,672,681 | ^658^GDAGPOGPAGPAGPOGPIGNVGAOGPK |
| 2316.0487 | 52 |  |  | 195, 198,210,216 | ^193^GEOGPOGPAGAAGPAGNOGADGQOGAK |
| 2323.1313 | 79 |  | *683* | 663,672,681 | ^658^GDAGPOGPAGPAGPOGPIGNVGAOG*OK* |
| 2332.0436 | 65 |  | 206 | 195,198,210,216 | ^193^GEOGPOGPAGAAGOAGNOGADGQOGAK |
| 2548.2063 | 101 |  |  | 156,165,168 | ^145^GNDGAVGAAGPOGPTGPTGPOGFOGAAGAK |
| 2564.2012 | 54 |  | 155 | 156,165,168 | ^145^GNDGAVGAAGOOGPTGPTGPOGFOGAAGAK |
| 2679.2394 | 42 | 948 |  | 942,945 | ^934^GFSGLQGPOGSOGSPGEQGPSGASGPAGPR |
| 2695.2343 | 132 |  |  | 942,945,948 | ^934^GFSGLQGPOGSOGSOGEQGPSGASGPAGPR |

**S2 Table: α1(I) chain of rat tail tendon, commercial sample, second trial (RC1A1_b)**

| **m/z ^+1^** | **Score** | | **P_y_** | | **O_x_** | **O_y_** | **Peptide** |
| --- | --- | --- | --- | --- | --- | --- | --- |
| 783.4359 | 52 | |  | |  | 561 | ^556^GAAGLOGPK |
| 836.4373 | 72 | |  | |  |  | ^907^GPAGPQGPR |
| 851.4258 | 46 | |  | |  |  | ^91^GFSGLDGAK |
| 852.4322 | 41 | |  | |  | 69 | ^67^GPOGPQGAR |
| 886.4377 | 63 | |  | |  |  | ^184^GSEGPQGVR |
| 898.5105 | 60 | |  | |  | 786 | ^781^GVVGLOGQR |
| 1088.5371 | 66 | |  | |  | 318 | ^316^GFOGADGVAGPK |
| 1105.5749 | 47 | |  | |  | 513 | ^508^GVQGPOGPAGPR |
| 1161.5721 | 67 | |  | |  | 405 | ^397^GQAGVMGFOGPK |
| 1177.5670 | 48 | |  | |  | 405 | ^397^GQAGVMGFOGPK |
| 1192.6321 | 74 | |  | |  | 423,426 | ^421^GVOGPOGAVGPAGK |
| 1258.5910 | 64 | |  | |  | 366,369 | ^361^GLTGSOGSOGPDGK |
| 1306.6386 | 82 | |  | |  | 249 | ^238^GPSGPQGPSGAOGPK |
| 1328.6481 | 76 | | 804 | |  | 795,798 | ^793^GFOGLOGPSGEPGK |
| 1338.6284 | 58 | |  | | 239,245 | 249 | ^238^GOSGPQGOSGAOGPK |
| 1344.6430 | 41 | |  | |  | 795,798,804 | ^793^GFOGLOGPSGEOGK |
| 1435.6812 | 43 | | 297 | |  | 303,306 | ^295^GEPGPSGLOGPOGER |
| 1443.6975 | 127 | |  | |  | 693,699 | ^688^GAAGPOGATGFOGAAGR |
| 1532.7816 | 67 | |  | |  |  | ^889^GETGPAGPAGPIGPAGAR |
| 1680.7572 | 86 | |  | |  | 444 | ^435^DGEAGAQGAOGPAGPAGER |
| 1744.7733 | 46 | |  | |  | 840,846,852 | ^837^EGSOGAEGSOGRDGAOGAK |
| 1819.8457 | 91 | |  | |  | 273,282 | ^271^GEOGPAGVQGPOGPAGEEGK |
| 1856.9137 | 44 | |  | | 707 | 708,717,720 | ^705^VGOOGPSGNAGPOGPOGPVGK |
| 1872.9086 | 49 | |  | | 707, 722 | 708,717,720 | ^705^VGOOGPSGNAGPOGPOGOVGK |
| 1959.9519 | 87 | | 273 | |  | 282 | ^271^GEPGPAGVQGPOGPAGEEGKR |
| 1975.9468 | 94 | |  | |  | 273,282 | ^271^GEOGPAGVQGPOGPAGEEGKR |
| 2014.9689 | 41 | |  | |  | 378,387,393 | ^375^TGPOGPAGQDGROGPAGPOGAR |
| 2030.9639 | 50 | |  | | 377 | 378,387,393 | ^375^TGOOGPAGQDGROGPAGPOGAR |
| 2120.9955 | 98 | | 771 | |  | 759,768 | ^757^GSOGADGPAGSOGTPGPQGIAGQR |
| 2136.9905 | 84 | |  | |  | 759,768,771 | ^757^GSOGADGPAGSOGTOGPQGIAGQR |
| 2169.0571 | 57 | | 876 | |  | 867,870,873 | ^859^GETGPAGPOGAOGAOGAPGPVGPAGK |
| 2185.0520 | 45 | |  | |  | 867,870,873,876 | ^859^GETGPAGPOGAOGAOGAOGPVGPAGK |
| 2216.0578 | 57 | |  | |  | 741,747,750 | ^733^GETGPAGROGEVGPOGPOGPAGEK |
| 2228.9837 | 99 | |  | |  | 537,540,546,552 | ^532^GDTGAOGAOGSQGAOGLQGMOGER |
| 2316.0487 | 53 | |  | |  | 195,198,210,216 | ^193^GEOGPOGPAGAAGPAGNOGADGQOGAK |
| 2332.0436 | 79 | |  | | 206 | 195,198,210,216 | ^193^GEOGPOGPAGAAGOAGNOGADGQOGAK |
| 2513.2015 | 142 | |  | |  | 867,870,873,876 | ^856^GDRGETGPAGPOGAOGAOGAOGPVGPAGK |
| 2548.2063 | 72 | |  | |  | 156,165,168 | ^145^GNDGAVGAAGPOGPTGPTGPOGFOGAAGAK |
| 2564.2012 | 69 | |  | | 155 | 156,165,168 | ^145^GNDGAVGAAGOOGPTGPTGPOGFOGAAGAK |
| 2679.2394 | 68 | | 948 | |  | 942,945 | ^934^GFSGLQGPOGSOGSPGEQGPSGASGPAGPR |
| 2695.2343 | 127 | |  | |  | 942,945,948 | ^934^GFSGLQGPOGSOGSOGEQGPSGASGPAGPR |
| **S3 Table: α1(I) chain of rat tail tendon, single rat tail tendon (srtt) sample (RC1α1_c)** | | | | | | | |
| **m/z^+1^** | **Score** | **P_y_** | | **O_x_** | | **O_y_** | **Peptide** |
| 783.4359 | 47 |  | |  | | 561 | ^556^GAAGLOGPK |
| 836.4373 | 44 |  | |  | |  | ^907^GPAGPQGPR |
| 851.4258 | 60 |  | |  | |  | ^91^GFSGLDGAK |
| 852.4322 | 43 |  | |  | | 69 | ^67^GPOGPQGAR |
| 886.4377 | 62 |  | |  | |  | ^184^GSEGPQGVR |
| 898.5105 | 56 |  | |  | | 786 | ^781^GVVGLOGQR |
| 943.4480 | 41 |  | |  | | 966,972 | ^964^GPOGSAGSOGK |
| 945.4384 | 56 |  | |  | |  | ^807^QGPSGASGER |
| 1088.5371 | 62 |  | |  | | 318 | ^316^GFOGADGVAGPK |
| 1132.4865 | 53 |  | |  | | 840,846 | ^837^EGSOGAEGSOGR |
| 1161.5721 | 72 |  | |  | | 405 | ^397^GQAGVMGFOGPK |
| 1177.5670 | 44 |  | |  | | 405 | ^397^GQAGVMGFOGPK |
| 1193.5619 | 54 |  | |  | | 405 | ^397^GQAGVMGFOG*PK* |
| 1242.5961 | 63 | 369 | |  | | 366 | ^361^GLTGSOGSPGPDGK |
| 1322.6335 | 76 |  | | 239 | | 249 | ^238^GOSGPQGPSGAOGPK |
| 1338.6284 | 76 |  | | 239,251 | | 249 | ^238^GOSGPQGPSGAOGOK |
| 1344.6430 | 57 |  | |  | | 795,798,804 | ^793^GFOGLOGPSGEOGK |
| 1354.6233 | 49 |  | | 239,245,*251* | | 249 | ^238^GOSGPQGOSGAOG*OK* |
| 1443.6975 | 42 |  | |  | | 693,699 | ^688^GAAGPOGATGFOGAAGR |
| 1451.6761 | 46 |  | |  | | 297,303,306 | ^295^GEOGPSGLOGPOGER |
| 1452.7264 | 65 | -1 | |  | |  | ^-7^SAGVSVPGPMGPSGPR |
| 1468.7213 | 51 | -1 | |  | |  | ^-7^SAGVSVPGPMGPSGPR |
| 1484.7162 | 40 |  | |  | | -1 | ^-7^SAGVSVOGPMGPSGPR |
| 1532.7816 | 84 |  | |  | |  | ^889^GETGPAGPAGPIGPAGAR |
| 1561.7969 | 40 |  | | 986 | | 981,987 | ^975^DGLNGLOGPIGOOGPR |
| 1574.8173 | 86 | 600 | |  | | 594 | ^586^GLTGPIGPOGPAGAPGDK |
| 1585.7717 | 78 |  | |  | | 225,231,234 | ^220^GANGAOGIAGAOGFOGAR |
| 1590.8122 | 42 |  | |  | | 594,600 | ^586^GLTGPIGPOGPAGAOGDK |
| 1655.7984 | 60 |  | |  | | 345,351,357 | ^343^GSOGEAGROGEAGLOGAK |
| 1664.7623 | 88 | 444 | |  | |  | ^435^DGEAGAQGAPGPAGPAGER |
| 1680.7551 | 96 |  | |  | | 444 | ^435^DGEAGAQGAOGPAGPAGER |
| 1696.7521 | 92 |  | | 449 | | 444 | ^435^DGEAGAQGAOGPAGOAGER |
| 1800.8697 | 105 | 825,831 | |  | | 819 | ^817^GPOGPMGPPGLAGPPGESGR |
| 1803.8508 | 77 | 273 | |  | | 282 | ^271^GEPGPAGVQGPOGPAGEEGK |
| 1816.8647 | 125 | 825,831 | |  | | 819 | ^817^GPOGPMGPPGLAGPPGESGR |
| 1819.8457 | 82 |  | |  | | 273,282 | ^271^GEOGPAGVQGPOGPAGEEGK |
| 1872.9086 | 49 |  | | 707, *717 or* *719* | | 708,720 | ^705^VGOOGPSGNAG*POGOOGP*VGK |
| 1888.9035 | 48 |  | | 707,719,722 | | 708,717,720 | ^705^VGOOGPSGNAGPOGOOGOVGK |
| 1975.9468 | 69 |  | |  | | 273,282 | ^271^GEOGPAGVQGPOGPAGEEGKR |
| 2105.0006 | 78 | 759,771 | |  | | 768 | ^757^GSPGADGPAGSOGTPGPQGIAGQR |
| 2244.9786 | 98 |  | |  | | 537,540,546,552 | ^532^GDTGAOGAOGSQGAOGLQGMOGER |
| 2339.1262 | 40 |  | | 668,*683* | | 663,672,*681* | ^658^GDAGPOGPAGOAGPOGPIGNVGAOG*OK* |
| 2513.2015 | 67 |  | |  | | 867,870,873,876 | ^856^GDRGETGPAGPOGAOGAOGAOGPVGPAGK |
| 2548.2063 | 58 |  | |  | | 156,165,168 | ^145^GNDGAVGAAGPOGPTGPTGPOGFOGAAGAK |
| 2564.2012 | 96 |  | | 155 | | 156,165,168 | ^145^GNDGAVGAAGOOGPTGPTGPOGFOGAAGAK |
| 2663.2444 | 96 | 945,948 | |  | | 942 | ^934^GFSGLQGPOGSPGSPGEQGPSGASGPAGPR |
| 2679.2394 | 105 | 948 | |  | | 942,945 | ^934^GFSGLQGPOGSOGSPGEQGPSGASGPAGPR |
| 2695.2343 | 62 |  | |  | | 942,945,948 | ^934^GFSGLQGPOGSOGSOGEQGPSGASGPAGPR |

**S4 Table: α2(I) chain of rat tail tendon, commercial sample, first trial (RC1α2_a)**

| **m/z ^+1^** | **Score** | **Py** | **Ox** | **Oy** | **Peptide** |
| --- | --- | --- | --- | --- | --- |
| 758.3791 | 56 |  |  | 318 | ^316^GLOGADGR |
| 868.4635 | 50 |  |  | 138 | ^135^VGAOGPAGAR |
| 868.4635 | 58 |  |  |  | ^907^GPSGPQGIR |
| 937.5102 | 41 |  |  |  | ^964^GPAGPSGPIGK |
| 987.4676 | 54 |  |  | 330 | ^324^AGVMGPOGNR |
| 1084.5633 | 71 |  |  | 258 | ^253^GLVGEOGPAGSK |
| 1159.5742 | 82 |  |  | 405 | ^397^GEAGNIGFOGPK |
| 1203.6229 | 48 |  |  | 981 | ^978^SGHOGPVGPAGVR |
| 1238.6052 | 57 |  |  | 78,81,84 | ^76^GFOGTOGLOGFK |
| 1239.6328 | 80 |  |  | 363,366 | ^361^GLOGSOGNVGPAGK |
| 1251.6076 | 77 |  |  |  | ^604^GEAGAAGPSGPAGPR |
| 1293.6910 | 83 |  |  | 240 | ^238^GIOGPVGAAGATGPR |
| 1399.6821 | 43 |  |  | -1 | ^-6^GVSAGOGPMGLMGPR |
| 1453.7434 | 89 |  |  | 486,492 | ^484^GLOGEFGLOGPAGPR |
| 1490.7122 | 49 |  |  | 750 | ^741^TGEIGASGPOGFAGEK |
| 1510.7245 | 68 |  |  | 588,594 | ^586^GAOGAIGAOGPAGASGDR |
| 1560.8129 | 102 | 891 |  |  | ^889^GEPGPAGSVGPVGAVGPR |
| 1576.8078 | 59 |  |  | 891 | ^889^GEOGPAGSVGPVGAVGPR |
| 1592.8391 | 83 |  |  | 225,231,234 | ^220^GATGLOGVAGAOGLOGPR |
| 1592.7663 | 58 | 297 |  | 294,306 | ^292^GSOGEPGSAGPAGPOGLR |
| 1608.7612 | 67 |  |  | 294,297,306 | ^292^GSOGEOGSAGPAGPOGLR |
| 1615.8187 | 81 |  |  | 183 | ^175^GELGPVGNOGPAGPAGPR |
| 1781.7937 | 69 | 273 |  | 282 | ^271^GEPGSAGAQGPOGPSGEEGK |
| 2098.104 | 52 |  |  | 381,387 | ^375^EGPVGLOGIDGROGPIGPAGPR |
| 2276.1054 | 122 |  |  | 861,873 | ^859^GYOGNIGPTGAAGAOGPHGSVGPAGK |
| 2384.0597 | 51 |  |  | 273,282 | ^265^GETGNKGEOGSAGAQGPOGPSGEEGK |
| 2605.2529 | 102 |  | *173* | 165,168,*171* | ^145^GSDGSVGPVGPAGPIGSAGPOGFOGAOG*OK* |
| 2743.3143 | 79 |  |  | 588,594, | ^586^GAOGAIGAOGPAGASGDRGEAGAAGPSGPAGPR |
| 2836.3496 | 73 |  |  | 462,468,480 | ^454^GEQGPAGPOGFQGLOGPSGTAGEVGKOGER |
| 2971.5120 | 76 | 762 |  | 768,780,786 | ^757^GPSGEPGTTGPOGTAGPQGLLGAOGILGLOGSR |
| 2987.5069 | 48 |  |  | 762,768,780,786 | ^757^GPSGEOGTTGPOGTAGPQGLLGAOGILGLOGSR |
| 3061.4206 | 71 |  |  | 525,540,552 | ^520^GPSGAOGPDGNKGEAGAVGAOGSAGASGPGGLOGER |
| 4202.0537 | 118 |  | 173 | 165,168,171,183 | ^145^GSDGSVGPVGPAGPIGSAGPOGFOGAOGOKGELGPVGNOGPAGPAGPR |
| **S5 Table: α2(I) chain of rat tail tendon, commercial sample, second trial (RC1A2_b)** | | | | | |

| **m/z ^+1^** | **Score** | **Py** | | **Ox** | **Oy** | **Peptide** |
| --- | --- | --- | --- | --- | --- | --- |
| 840.4686 | 60 |  | |  |  | ^67^GVVGPQGAR |
| 868.4635 | 71 |  | |  | 138 | ^135^VGAOGPAGAR |
| 868.4635 | 46 |  | |  |  | ^907^GPSGPQGIR |
| 987.4676 | 52 |  | |  | 330 | ^324^AGVMGPOGNR |
| 1057.5636 | 42 |  | |  | 561 | ^556^GAAGIOGGKGEK |
| 1084.5633 | 75 |  | |  | 258 | ^253^GLVGEOGPAGSK |
| 1159.5742 | 52 |  | |  | 405 | ^397^GEAGNIGFOGPK |
| 1238.6052 | 58 |  | |  | 78,81,84 | ^76^GFOGTOGLOGFK |
| 1239.6328 | 50 |  | |  | 363,366 | ^361^GLOGSOGNVGPAGK |
| 1251.6076 | 81 |  | |  |  | ^604^GEAGAAGPSGPAGPR |
| 1293.6910 | 94 |  | |  | 240 | ^238^GIOGPVGAAGATGPR |
| 1399.6821 | 65 | -1 | |  |  | ^-6^GVSAGPGPMGLMGPR |
| 1453.7434 | 87 |  | |  | 486,492 | ^484^GLOGEFGLOGPAGPR |
| 1510.7245 | 81 |  | |  | 588,594 | ^586^GAOGAIGAOGPAGASGDR |
| 1560.8129 | 111 | 891 | |  |  | ^889^GEPGPAGSVGPVGAVGPR |
| 1576.8078 | 91 |  | |  | 891 | ^889^GEOGPAGSVGPVGAVGPR |
| 1576.7714 | 79 | 294,297 | |  | 306 | ^292^GSPGEPGSAGPAGPOGLR |
| 1592.7663 | 50 | 297 | |  | 294,306 | ^292^GSOGEPGSAGPAGPOGLR |
| 1592.8391 | 88 |  | |  | 225,231,234 | ^220^GATGLOGVAGAOGLOGPR |
| 1608.7612 | 76 |  | |  | 294,297,306 | ^292^GSOGEOGSAGPAGPOGLR |
| 1615.8187 | 93 |  | |  | 183 | ^175^GELGPVGNOGPAGPAGPR |
| 1781.7937 | 69 | 273 | |  | 282 | ^271^GEPGSAGAQGPOGPSGEEGK |
| 1833.8977 | 47 | 717 | | 707,719 | 708,720 | ^705^TGOOGPSGITGPPGOOGAAGK |
| 1892.8118 | 45 |  | |  | 840,846,852 | ^837^DGNOGSDGPOGRDGQOGHK |
| 1937.8948 | 115 | 273 | |  | 282 | ^271^GEPGSAGAQGPOGPSGEEGKR |
| 2041.0322 | 97 |  | |  | 891 | ^885^HGNRGEOGPAGSVGPVGAVGPR |
| 2098.1040 | 56 |  | |  | 381,387 | ^375^EGPVGLOGIDGROGPIGPAGPR |
| 2276.1054 | 134 |  | |  | 861,873 | ^859^GYOGNIGPTGAAGAOGPHGSVGPAGK |
| 2368.0648 | 41 | 273 | |  | 282 | ^265^GETGNKGEPGSAGAQGPOGPSGEEGK |
| 2384.0597 | 60 |  | |  | 273,282 | ^265^GETGNKGEOGSAGAQGPOGPSGEEGK |
| 2605.2529 | 80 |  | | 173 | 165,168,171 | ^145^GSDGSVGPVGPAGPIGSAGPOGFOGAOGOK |
| 2743.3143 | 88 |  | |  | 588,594 | ^586^GAOGAIGAOGPAGASGDRGEAGAAGPSGPAGPR |
| 2836.3496 | 79 |  | |  | 462,468,480 | ^454^GEQGPAGPOGFQGLOGPSGTAGEVGKOGER |
| 3061.4206 | 72 |  | |  | 525,540,552 | ^520^GPSGAOGPDGNKGEAGAVGAOGSAGASGPGGLOGER |
| **S6 Table: α2(I) chain of rat tail tendon, single rat tail (srtt) sample (RC1A2_c)** | | | | | | |
| **m/z^+1^** | **Score** | **Py** | **Ox** | | **Oy** | **Peptide** |
| 758.3791 | 48 |  |  | | 318 | ^316^GLOGADGR |
| 840.4686 | 54 |  |  | |  | ^67^GVVGPQGAR |
| 868.4635 | 62 |  |  | | 138 | ^135^VGAOGPAGAR |
| 868.4653 | 51 |  |  | |  | ^907^GPSGPQGIR |
| 884.4585 | 46 |  | 908 | |  | ^907^GOSGPQGIR |
| 937.5102 | 52 |  |  | |  | ^964^GPAGPSGPIGK |
| 953.5051 | 51 |  | 968 | |  | ^964^GPAGOSGPIGK |
| 971.4727 | 44 |  |  | | 330 | ^324^AGVMGPOGNR |
| 987.4676 | 54 |  |  | | 330 | ^324^AGVMGPOGNR |
| 1068.5684 | 71 | 258 |  | |  | ^253^GLVGEPGPAGSK |
| 1084.5633 | 70 |  |  | | 258 | ^253^GLVGEOGPAGSK |
| 1157.4818 | 57 |  |  | | 840,846 | ^837^DGNOGSDGPOGR |
| 1175.5691 | 79 |  | *407* | | 405 | ^397^GEAGNIGFOG*PK* |
| 1187.6280 | 54 | 981 |  | |  | ^978^SGHPGPVGPAGVR |
| 1238.6052 | 64 |  |  | | 78,81,84 | ^76^GFOGTOGLOGFK |
| 1239.6328 | 85 |  |  | | 363,636 | ^361^GLOGSOGNVGPAGK |
| 1251.6076 | 77 |  |  | |  | ^604^GEAGAAGPSGPAGPR |
| 1399.6821 | 95 |  |  | |  | ^-6^GVSAGPGPMGLMGPR |
| 1415.6770 | 87 |  |  | |  | ^-6^GVSAGPGPMGLMGPR |
| 1428.7190 | 52 |  |  | | 579 | ^568^GETGLRGEIGNOGR |
| 1431.6719 | 46 |  |  | | *-1* | ^-6^GVSAG*O*GPMGLMG*P*R |
| 1437.7485 | 79 | 486 |  | | 492 | ^484^GLPGEFGLOGPAGPR |
| 1453.7434 | 75 |  |  | | 486,492 | ^484^GLOGEFGLOGPAGPR |
| 1469.7383 | 110 |  | 494 | | 486,492 | ^484^GLOGEFGLOGOAGPR |
| 1490.7122 | 59 |  |  | | 750 | ^741^TGEIGASGPOGFAGEK |
| 1506.7071 | 67 |  | 749 | | 750 | ^741^TGEIGASGOOGFAGEK |
| 1510.7245 | 86 |  |  | | 588,594 | ^586^GAOGAIGAOGPAGASGDR |
| 1533.6751 | 74 |  |  | | 702,708 | ^697^GDGGPOGMTGFOGAAGR |
| 1560.8129 | 102 | 891 |  | |  | ^889^GEPGPAGSVGPVGAVGPR |
| 1576.8078 | 110 |  |  | | 891 | ^889^GEOGPAGSVGPVGAVGPR |
| 1576.7714 | 76 | 294,297 |  | | 306 | ^292^GSPGEPGSAGPAGPOGLR |
| 1592.7663 | 85 | 294 |  | | 297,306 | ^292^GSPGEOGSAGPAGPOGLR |
| 1599.8238 | 87 | 183 |  | |  | ^175^GELGPVGNPGPAGPAGPR |
| 1608.7612 | 88 |  |  | | 294,297,306 | ^292^GSOGEOGSAGPAGPOGLR |
| 1615.8187 | 105 |  |  | | 183 | ^175^GELGPVGNOGPAGPAGPR |
| 1624.7562 | 94 |  | 302 | | 294,297,306 | ^292^GSOGEOGSAGOAGPOGLR |
| 1748.8674 | 69 | 297 |  | | 294,306 | ^291^RGSOGEPGSAGPAGPOGLR |
| 1751.8307 | 107 |  |  | | 819,825,831 | ^817^GPOGAVGSOGVNGAOGEAGR |
| 1764.8624 | 50 |  |  | | 294,297,306 | ^291^RGSOGEOGSAGPAGPOGLR |
| 1781.7937 | 85 | 273 |  | | 282 | ^271^GEPGSAGAQGPOGPSGEEGK |
| 1797.7886 | 94 |  |  | | 273,282 | ^271^GEOGSAGAQGPOGPSGEEGK |
| 1817.9028 | 52 |  |  | | 708,717,720 | ^705^TGPOGPSGITGPOGPOGAAGK |
| 1833.8977 | 69 |  | 707 | | 708, 717,720 | ^705^TGOOGPSGITGPOGPOGAAGK |
| 1849.8926 | 89 |  | 707,716 | | 708,717,720 | ^705^TGOOGPSGITGOOGPOGAAGK |
| 1865.8876 | 97 |  | 707,716,719 | | 708,717,720 | ^705^TGOOGPSGITGOOGOOGAAGK |
| 1953.8897 | 52 |  |  | | 273,282 | ^271^GEOGSAGAQGPOGPSGEEGKR |
| 2010.9475 | 86 |  |  | | 540,552 | ^532^GEAGAVGAOGSAGASGPGGLOGER |
| 2025.0373 | 82 | 891 |  | |  | ^885^HGNRGEPGPAGSVGPVGAVGPR |
| 2041.0322 | 76 |  |  | | 891 | ^885^HGNRGEOGPAGSVGPVGAVGPR |
| 2098.1040 | 53 |  |  | | 381,387 | ^375^EGPVGLOGIDGROGPIGPAGPR |
| 2145.9908 | 76 |  |  | | 432,444 | ^430^GAOGPDGNNGAQGPOGPQGVQGGK |
| 2368.0648 | 61 | 273 |  | | 282 | ^265^GETGNKGEPGSAGAQGPOGPSGEEGK |
| 2524.1659 | 54 | 273 |  | | 282 | ^265^GETGNKGEPGSAGAQGPOGPSGEEGKR |
| 2540.1608 | 52 |  |  | | 273,282 | ^265^GETGNKGEOGSAGAQGPOGPSGEEGKR |
| 2589.2580 | 112 |  |  | | 165,168,171 | ^145^GSDGSVGPVGPAGPIGSAGPOGFOGAOGPK |
| 2605.2529 | 103 |  | *173* | | 165,168,171 | ^145^GSDGSVGPVGPAGPIGSAGPOGFOGAOG*OK* |
| 2621.2478 | 51 |  | 164, *173* | | 165,168,171 | ^145^GSDGSVGPVGPAGPIGSAGOOGFOGAOG*OK* |
| 2786.4081 | 77 |  |  | | 952,964 | ^934^GHNGLQGLOGLAGLHGDQGAOGPVGPAGPR |
| 2987.5069 | 84 |  |  | | 762,768,780,786 | ^757^GPSGEOGTTGPOGTAGPQGLLGAOGILGLOGSR |
| 3045.4257 | 78 | 552 |  | | 525,540 | ^520^GPSGAOGPDGNKGEAGAVGAOGSAGASGPGGLPGER |
| 3077.4155 | 76 |  | 548 | | 525,540,552 | ^520^GPSGAOGPDGNKGEAGAVGAOGSAGASGOGGLOGER |
| 4186.0588 | 64 | 183 | 173 | | 165,168,171 | ^145^GSDGSVGPVGPAGPIGSAGPOGFOGAOGOKGELGPVGNPGPAGPAGPR |
| 4218.0487 | 80 |  | 164,173 | | 165,168,171.183 | ^145^GSDGSVGPVGPAGPIGSAGOOGFOGAOGOKGELGPVGNOGPAGPAGPR |
| 4459.2012 | 41 |  |  | | 750,762,768,780,786 | ^741^TGEIGASGPOGFAGEKGPSGEOGTTGPOGTAGPQGLLGAOGILGLOGSR |

**S7 Table: human α1(I), first trial (HC1A1_a)**

| **m/z ^+1^** | **Score** | **Py** | **Ox** | **Oy** | **Peptide** |
| --- | --- | --- | --- | --- | --- |
| 836.4373 | 69 |  |  |  | ^907^GPAGPQGPR |
| 886.4377 | 50 |  |  |  | ^184^GSEGPQGVR |
| 898.5105 | 58 |  |  | 786 | ^781^GVVGLOGQR |
| 1088.5371 | 48 |  |  | 318 | ^316^GFOGADGVAGPK |
| 1105.5749 | 40 |  |  | 513 | ^508^GVQGPOGPAGPR |
| 1177.5670 | 49 |  |  | 405 | ^397^GQAGVMGFOGPK |
| 1258.5910 | 69 |  |  | 366,369 | ^361^GLTGSOGSOGPDGK |
| 1302.6437 | 80 |  |  | 249 | ^238^GPSGPQGPGGPOGPK |
| 1344.6430 | 48 |  |  | 795,798,804 | ^793^GFOGLOGPSGEOGK |
| 1459.6924 | 135 |  |  | 693,699 | ^688^GSAGPOGATGFOGAAGR |
| 1465.6918 | 74 |  |  | 297,303,306 | ^295^GEOGPTGLOGPOGER |
| 1546.7972 | 76 |  |  |  | ^889^GETGPAGPAGPVGPVGAR |
| 1706.7729 | 104 |  |  | 444 | ^435^DGEAGAQGPOGPAGPAGER |
| 1812.8875 | 49 |  |  | 708,717,720 | ^705^VGPOGPSGNAGPOGPOGPAGK |
| 1832.8596 | 78 | 825 |  | 819,831 | ^817^GPOGPMGPPGLAGPOGESGR |
| 1848.8545 | 41 |  |  | 819,825,831 | ^817^GPOGPMGPOGLAGPOGESGR |
| 2003.9781 | 100 |  |  | 273,282 | ^271^GEOGPVGVQGPOGPAGEEGKR |
| 2056.9795 | 67 |  |  | 378,387,390,393 | ^375^TGPOGPAGQDGROGPOGPOGAR |
| 2105.0006 | 83 | 771 |  | 759,768 | ^757^GSOGADGPAGAOGTPGPQGIAGQR |
| 2149.9785 | 73 |  |  | 627, 630,639,645 | ^625^GEOGPOGPAGFAGPOGADGQOGAK |
| 2281.1207 | 61 |  |  | 663,672,681 | ^658^GDAGPOGPAGPAGPOGPIGNVGAOGAK |
| 2316.0487 | 97 |  |  | 195, 198,210,216 | ^193^GEOGPOGPAGAAGPAGNOGADGQOGAK |
| 2497.2066 | 72 | 876 |  | 867,870,873 | ^856^GDRGETGPAGPOGAOGAOGAPGPVGPAGK |
| 2703.2394 | 40 | 645 |  | 621,627, 630,639 | ^619^GAOGDRGEOGPOGPAGFAGPOGADGQPGAK |
| 2705.2550 | 132 |  |  | 942,945,948 | ^934^GFSGLQGPOGPOGSOGEQGPSGASGPAGPR |
| 2869.4075 | 73 |  |  | 594,600 | ^586^GLTGPIGPOGPAGAOGDKGESGPSGPAGPTGAR |
| 4112.9180 | 68 |  |  | 462,468,474,480,486,492 | ^454^GEQGPAGSOGFQGLOGPAGPOGEAGKOGEQGVOGDLGAOGPSGAR |
|  |  |  |  |  |  |
|  |  |  |  |  |  |

**S9 Table: human α1(I), second trial (HC1A1_b)**

| **m/z ^+1^** | **Score** | **Py** | **Ox** | **Oy** | **Peptide** |
| --- | --- | --- | --- | --- | --- |
| 836.4373 | 67 |  |  |  | ^907^GPAGPQGPR |
| 886.4377 | 58 |  |  |  | ^184^GSEGPQGVR |
| 1192.6321 | 71 |  |  |  | ^421^GVOGPOGAVGPAGK |
| 1459.6924 | 101 |  |  | 693, 699 | ^688^GSAGPOGATGFOGAAGR |
| 1465.6918 | 78 |  |  | 297,303,306 | ^295^GEOGPTGLOGPOGER |
| 1546.7972 | 67 |  |  |  | ^889^GETGPAGPAGPVGPVGAR |
| 1561.7969 | 65 |  | 986 | 981,987 | ^975^DGLNGLOGPI**G**OOGPR |
| 1585.7717 | 72 |  |  |  | ^220^GANGAOGIAGAOGFOGAR |
| 1706.7729 | 93 |  |  | 444 | ^435^DGEAGAQGPOGPAGPAGER |
| 1758.7348 | 79 |  |  | 111, 114,120 | ^109^GEOGSOGENGAOGQMGPR |
| 2003.9781 | 72 |  |  | 273,282 | ^271^GEOGPVGVQGPOGPAGEEGKR |
| 2105.0006 | 66 | 771 |  | 759,768 | ^757^GSOGADGPAGAOGTPGPQGIAGQR |
| 2120.9955 | 58 |  |  | 759,768,771 | ^757^GSOGADGPAGAOGTOGPQGIAGQR |
| 2149.9785 | 57 |  |  | 627, 630,639,645 | ^625^GEOGPOGPAGFAGPOGADGQOGAK |
| 2281.1207 | 84 |  |  | 663,672,681 | ^658^GDAGPOGPAGPAGPOGPIGNVGAOGAK |
| 2497.2066 | 81 | 876 |  | 867,870,873 | ^856^GDRGETGPAGPOGAOGAOGAPGPVGPAGK |
| 2513.2015 | 109 |  |  | 867,870,873,876 | ^856^GDRGETGPAGPOGAOGAOGAOGPVGPAGK |
| 2703.2394 | 63 | 645 |  | 621,627, 630,639 | ^619^GAOGDRGEOGPOGPAGFAGPOGADGQPGAK |
| 2705.2550 | 111 |  |  | 942,945,948 | ^934^GFSGLQGPOGPOGSOGEQGPSGASGPAGPR |
| 2869.4075 | 97 |  |  | 594,600 | ^586^GLTGPIGPOGPAGAOGDKGESGPSGPAGPTGAR |

**S9 Table: human α2(I), second trial (HC1A2_a)**

| **m/z ^+1^** | **Score** | **Py** | **Ox** | **Oy** | **Peptide** |
| --- | --- | --- | --- | --- | --- |
| 809.4377 | 48 |  |  |  | ^421^GHAGLAGAR |
| 868.4635 | 73 |  |  | 138 | ^135^VGAOGPAGAR |
| 868.4635 | 40 |  |  |  | ^907^GPSGPQGIR |
| 895.4632 | 54 |  |  |  | ^964^GPAGPSGPAGK |
| 960.4567 | 62 |  |  | 330 | ^324^AGVMGPOGSR |
| 1084.5633 | 42 |  |  | 258 | ^253^GLVGEOGPAGSK |
| 1201.5848 | 67 |  |  | 399,405 | ^397^GEOGNIGFOGPK |
| 1253.6484 | 89 |  |  | 363,366 | ^361^GLOGSOGNIGPAGK |
| 1267.6753 | 51 |  |  | 240 | ^238^GIOGPVGAAGATGAR |
| 1427.7134 | 56 |  |  |  | ^-6^GVGLGPGPMGLMGPR |
| 1477.7546 | 67 |  |  | 492 | ^484^GLHGEFGLOGPAGPR |
| 1488.7329 | 49 |  |  | 750 | ^741^TGEVGAVGPOGFAGEK |
| 1510.7245 | 69 |  |  | 588,594 | ^586^GAOGAVGAOGPAGATGDR |
| 1549.6700 | 80 |  |  | 693, 699 | ^688^GDGGPOGMTGFOGAAGR |
| 1562.7921 | 85 |  |  |  | ^889^GETGPSGPVGPAGAVGPR |
| 1580.7663 | 81 |  |  | 504 | ^502^GPOGESGAAGPTGPIGSR |
| 2027.0166 | 119 |  |  |  | ^885^HGNRGETGPSGPVGPAGAVGPR |
| 2115.1193 | 134 |  |  | 795,804,813 | ^793^GLOGVAGAVGEOGPLGIAGPOGAR |
| 2284.1469 | 106 |  |  | 861,873 | ^859^GYOGNIGPVGAAGAOGPHGPVGPAGK |
| 2727.3193 | 88 |  |  | 588,594 | ^586^GAOGAVGAOGPAGATGDRGEAGAAGPAGPAGPR |
| 2957.4963 | 76 |  |  | 768,771,780,786 | ^757^GPSGEAGTAGPOGTOGPQGLLGAOGILGLOGSR |
| 2959.3565 | 47 |  |  | 12,18,30,33 | ^10^GPOGAAGAOGPQGFQGPAGEOGEOGQTGPAGAR |

**S10 Table: human α2(I), first trial (HC1A2_b)**

| **m/z ^+1^** | **Score** | **Py** | **Ox** | **Oy** | **Peptide** |
| --- | --- | --- | --- | --- | --- |
| 868.4635 | 78 |  |  | 138 | ^135^VGAOGPAGAR |
| 895.4632 | 65 |  |  |  | ^964^GPAGPSGPAGK |
| 1168.4978 | 61 | 840 |  | 846 | ^837^DGNPGNDGPOGR |
| 1201.5848 | 59 |  |  | 399,405 | ^397^GEOGNIGFOGPK |
| 1267.6753 | 96 |  |  | 240 | ^238^GIOGPVGAAGATGAR |
| 1477.7546 | 78 |  |  | 492 | ^484^GLHGEFGLOGPAGPR |
| 1580.7663 | 75 |  |  | 504 | ^502^GPOGESGAAGPTGPIGSR |
| 1619.7772 | 102 |  |  | 303,306 | ^292^GPNGEAGSAGPOGPOGLR |
| 1775.8783 | 77 |  |  | 303,306 | ^291^RGPNGEAGSAGPOGPOGLR |
| 1823.8042 | 89 |  |  | 273,282 | ^271^GEOGSAGPQGPOGPSGEEGK |
| 2027.0166 | 89 |  |  |  | ^885^HGNRGETGPSGPVGPAGAVGPR |
| 2050.9941 | 66 |  |  | 645 | ^625^GEVGPAGPNGFAGPAGAAGQOGAK |
| 2115.1193 | 124 |  |  | 795,804,813 | ^793^GLOGVAGAVGEOGPLGIAGPOGAR |
| 2284.1469 | 106 |  |  | 861,873 | ^859^GYOGNIGPVGAAGAOGPHGPVGPAGK |
| 2396.0597 | 93 |  |  | 273,282 | ^265^GESGNKGEOGSAGPQGPOGPSGEEGK |
| 3397.7108 | 67 |  |  | 930,942,954 | ^928^GLOGLKGHNGLQGLOGIAGHHGDQGAOGSVGPAGPR |

**S11 Table: human α1(III), first trial (**HC3A1_a)

| **m/z ^+1^** | **Score** | **Py** | **Ox** | **Oy** | **Peptide** |
| --- | --- | --- | --- | --- | --- |
| 949.5102 | 47 | 981 |  |  | ^973^GPVGPSGPPGK |
| 965.5051 | 73 |  |  | 981 | ^973^GPVGPSGPOGK |
| 1094.5953 | 62 |  |  | 519,525 | ^517^GPOGLAGAOGLR |
| 1154.5623 | 41 |  |  | 795,798 | ^790^GLAGPOGMOGPR |
| 1173.5131 | 69 |  |  | 849,855 | ^846^DGNOGSDGLOGR |
| 1207.5702 | 76 |  |  | 432,435,441 | ^430^GGOGGOGPQGPOGK |
| 1303.6026 | 48 |  |  | 699,702,708 | ^697^GSOGGOGAAGFOGAR |
| 1508.7088 | 51 |  |  | 909 | ^898^GESGPAGPAGAOGPAGSR |
| 1530.7295 | 40 |  |  | 990,996 | ^984^DGTSGHOGPIGPOGPR |
| 1702.7887 | 58 |  |  | 237,240 | ^229^GEMGPAGIOGAOGLMGAR |
| 1833.9090 | 110 |  |  | 828,834,843 | ^826^GPOGPQGLOGLAGTAGEOGR |
| 1921.8886 | 92 |  |  | 738,747,750 | ^735^DGPOGPAGNTGAOGSOGVSGPK |
| 2104.0782 | 103 |  |  | 768,774,777 | ^766^GSOGAQGPOGAOGPLGIAGITGAR |
| 2180.0037 | 72 |  |  | 546,549,555,561 | ^541^GAAGPOGPOGAAGTOGLQGMOGER |
| 2283.1000 | 79 |  |  | 672,678,687 | ^667^GEGGPOGVAGPOGGSGPAGP*OGPQGVK* |
| 2641.2601 | 72 |  |  | 672,678,687 | ^667^GEGGPOGVAGPOGGSGPAGPOGPQGVKGER |
| 2690.2553 | 78 |  |  | 945,948,951,954,966 | ^943^GFOGNOGAOGSOGPAGQQGAIGSOGPAGPR |
| 2950.4653 | 81 |  |  | 603,609,618,621 | ^595^GPTGPIGPOGPAGQOGDKGEGGAOGLOGIAGPR |

**S12 Table: human α1(III), second trial (HC3A1_b)**

| **m/z ^+1^** | **Score** | **Py** | **Ox** | **Oy** | **Peptide** |
| --- | --- | --- | --- | --- | --- |
| 949.5102 | 67 | 981 |  |  | ^973^GPVGPSGPPGK |
| 965.5051 | 40 |  |  | 981 | ^973^GPVGPSGPOGK |
| 1094.5953 | 77 |  |  | 519,525 | ^517^GPOGLAGAOGLR |
| 1138.5674 | 65 |  |  | 795,798 | ^790^GLAGPOGMOGPR |
| 1173.5131 | 86 |  |  | 849,855 | ^846^DGNOGSDGLOGR |
| 1203.5827 | 49 |  |  | 408,414 | ^406^GQOGVMGFOGPK |
| 1207.5702 | 75 |  |  | 432,435,441 | ^430^GGOGGOGPQGPOGK |
| 1303.6026 | 57 |  |  | 699,702,708 | ^697^GSOGGOGAAGFOGAR |
| 1508.7088 | 69 |  |  | 909 | ^898^GESGPAGPAGAOGPAGSR |
| 1530.7295 | 55 |  |  | 990,996 | ^984^DGTSGHOGPIGPOGPR |
| 1631.8024 | 80 |  |  | 603,609 | ^595^GPTGPIGPOGPAGQOGDK |
| 1670.7989 | 72 |  |  | 237,240 | ^229^GEMGPAGIOGAOGLMGAR |
| 1833.9090 | 117 |  |  | 828,834,843 | ^826^GPOGPQGLOGLAGTAGEOGR |
| 1921.8886 | 90 |  |  | 738,747,750 | ^735^DGPOGPAGNTGAOGSOGVSGPK |
| 2104.0782 | 125 |  |  | 768,774,777 | ^766^GSOGAQGPOGAOGPLGIAGITGAR |
| 2164.0088 | 122 |  |  | 546,549,555,561 | ^541^GAAGPOGPOGAAGTOGLQGMOGER |
| 2283.1000 | 71 |  |  | 672,678 *687* | ^667^GEGGPOGVAGPOGGSGPAGP*OGPQGVK* |
| 2690.2553 | 94 |  |  | 945,948,951,954,966 | ^943^GFOGNOGAOGSOGPAGQQGAIGSOGPAGPR |
| 2950.4653 | 82 |  |  | 603,609,618,621 | ^595^GPTGPIGPOGPAGQOGDKGEGGAOGLOGIAGPR |

**S13 Table: human α1(III), third trial (HC3A1_c)**

| **m/z ^+1^** | **Score** | **Py** | **Ox** | **Oy** | **Peptide** |
| --- | --- | --- | --- | --- | --- |
| 949.5102 | 45 | 981 |  |  | ^973^GPVGPSGPPGK |
| 965.5051 | 44 |  |  | 981 | ^973^GPVGPSGPOGK |
| 1203.5827 | 57 |  |  | 408,414 | ^406^GQOGVMGFOGPK |
| 1219.5776 | 42 |  | *416* | 408,414 | ^406^GQOGVMGFOG*OK* |
| 1514.7346 | 50 | 990 |  | 996 | ^984^DGTSGHPGPIGPOGPR |
| 1530.7295 | 57 |  |  | 990,996 | ^984^DGTSGHOGPIGPOGPR |
| 1631.8024 | 96 |  |  | 603,609 | ^595^GPTGPIGPOGPAGQOGDK |
| 1670.7989 | 51 |  |  | 237,240 | ^229^GEMGPAGIOGAOGLMGAR |
| 1756.7733 | 88 |  |  | 303,306,312 | ^300^DGSOGEOGANGLOGAAGER |
| 1833.9090 | 125 |  |  | 828,834,843 | ^826^GPOGPQGLOGLAGTAGEOGR |
| 2088.0832 | 94 | 777 |  | 768,774 | ^766^GSOGAQGPOGAPGPLGIAGITGAR |
| 2104.0782 | 137 |  |  | 768,774,777 | ^766^GSOGAQGPOGAOGPLGIAGITGAR |
| 2164.0088 | 107 |  |  | 546,549,555,561 | ^541^GAAGPOGPOGAAGTOGLQGMOGER |
| 2180.0037 | 97 |  |  | 546,549,555,561 | ^541^GAAGPOGPOGAAGTOGLQGMOGER |
| 2283.1000 | 89 |  |  | 672,678,687 | ^667^GEGGPOGVAGPOGGSGPAGPOGPQGVK |
| 2299.0948 | 106 |  | *686* | 672,678,687 | ^667^GEGGPOGVAGPOGGSGPA*GOOGPQGVK* |
| 2690.2553 | 98 |  |  | 945,948,951,954,966 | ^943^GFOGNOGAOGSOGPAGQQGAIGSOGPAGPR |
| 2966.4603 | 58 |  | *605* | 603,609,618,621 | ^595^GPTGPIGPOG***O****AGQOGDK*GEGGAOGLOGIAGPR |

**S14 Table: human α1(III), forth trial (HC3α1_d)**

| **m/z ^+1^** | **Score** | **Py** | **Ox** | **Oy** | **Peptide** |
| --- | --- | --- | --- | --- | --- |
| 949.5102 | 42 | 981 |  |  | ^973^GPVGPSGPPGK |
| 965.5051 | 41 |  |  | 981 | ^973^GPVGPSGPOGK |
| 1094.5953 | 52 |  |  | 519,525 | ^517^GPOGLAGAOGLR |
| 1138.5674 | 44 |  |  | 795,798 | ^790^GLAGPOGMOGPR |
| 1173.5131 | 44 |  |  | 849,855 | ^846^DGNOGSDGLOGR |
| 1203.5827 | 52 |  |  | 408,414 | ^406^GQOGVMGFOGPK |
| 1303.6026 | 68 |  |  | 699,702,708 | ^697^GSOGGOGAAGFOGAR |
| 1337.6808 | 42 |  |  | 618,621 | ^613^GEGGAOGLOGIAGPR |
| 1508.7088 | 77 |  |  | 909 | ^898^GESGPAGPAGAOGPAGSR |
| 1530.7295 | 66 |  |  | 990,996 | ^984^DGTSGHOGPIGPOGPR |
| 1631.8024 | 90 |  |  | 603,609 | ^595^GPTGPIGPOGPAGQOGDK |
| 1670.7989 | 42 | 240 |  | 237 | ^229^GEMGPAGIOGAPGLMGAR |
| 1833.9090 | 136 |  |  | 828,834,843 | ^826^GPOGPQGLOGLAGTAGEOGR |
| 2088.0832 | 117 | 777 |  | 768,774 | ^766^GSOGAQGPOGAPGPLGIAGITGAR |
| 2104.0782 | 126 |  |  | 768,774,777 | ^766^GSOGAQGPOGAOGPLGIAGITGAR |
| 2164.0088 | 105 |  |  | 546,549,555,561 | ^541^GAAGPOGPOGAAGTOGLQGMOGER |
| 2196.9792 | 52 |  |  | 639,645,648,654 | ^634^GETGPOGPAGFOGAOGQNGEOGGK |
| 2283.1000 | 98 |  |  | 672,678,687 | ^667^GEGGPOGVAGPOGGSGPAGPOGPQGVK |
| 2299.0949 | 93 |  | *686* | 672,678,687 | ^667^GEGGPOGVAGPOGGSGPA*GOOGPQGVK* |
| 2690.2553 | 68 |  |  | 945,948,951,954,966 | ^943^GFOGNOGAOGSOGPAGQQGAIGSOGPAGPR |
| 2966.4603 | 60 |  | *605* | 603,609,618,621 | ^595^GPTGPIGPOG*OAGQOGDK*GEGGAOGLOGIAGPR |
