# Supplementary figures and images for: Heterogeneity in proline hydroxylation of fibrillar collagens observed by mass spectrometry
Source: PLoS One. 2021 Aug 31;16(8):e0250544. doi: 10.1371/journal.pone.0250544 (PMC8407550; doi:10.1371/journal.pone.0250544)

**A**

(Col III  $\alpha 1$ )<sub>2</sub>

Col III  $\alpha 1$  →

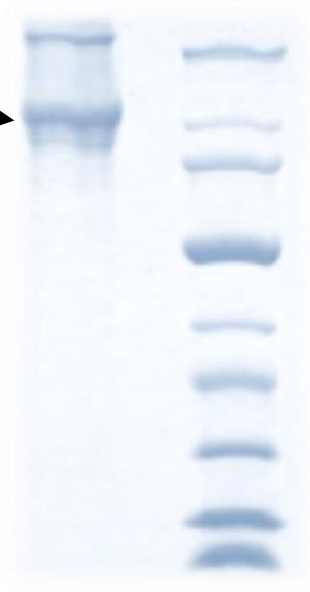

200,000

116,000

97,000

66,000

55,000

45,000

36,000

29,000

24,000

**B**

(Col I  $\alpha 1$ )<sub>2</sub>

Col I  $\alpha 1$  →

Col I  $\alpha 2$  →

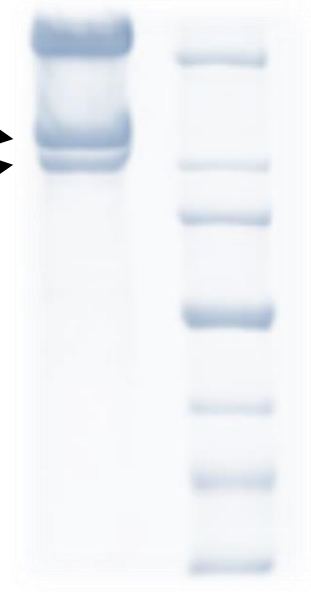

200,000

116,000

97,000

66,000

55,000

45,000

36,000

Supplement: S1 Fig — A: lane 1 –human collagen type III, lane 2 = molecular marker (Sigma). B: lane 1 = rat tail tendon collagen type I, lane 2 = molecular marker (Sigma). Gels were stained with coomassie blue. (PDF) [file pone.0250544.s002.pdf]

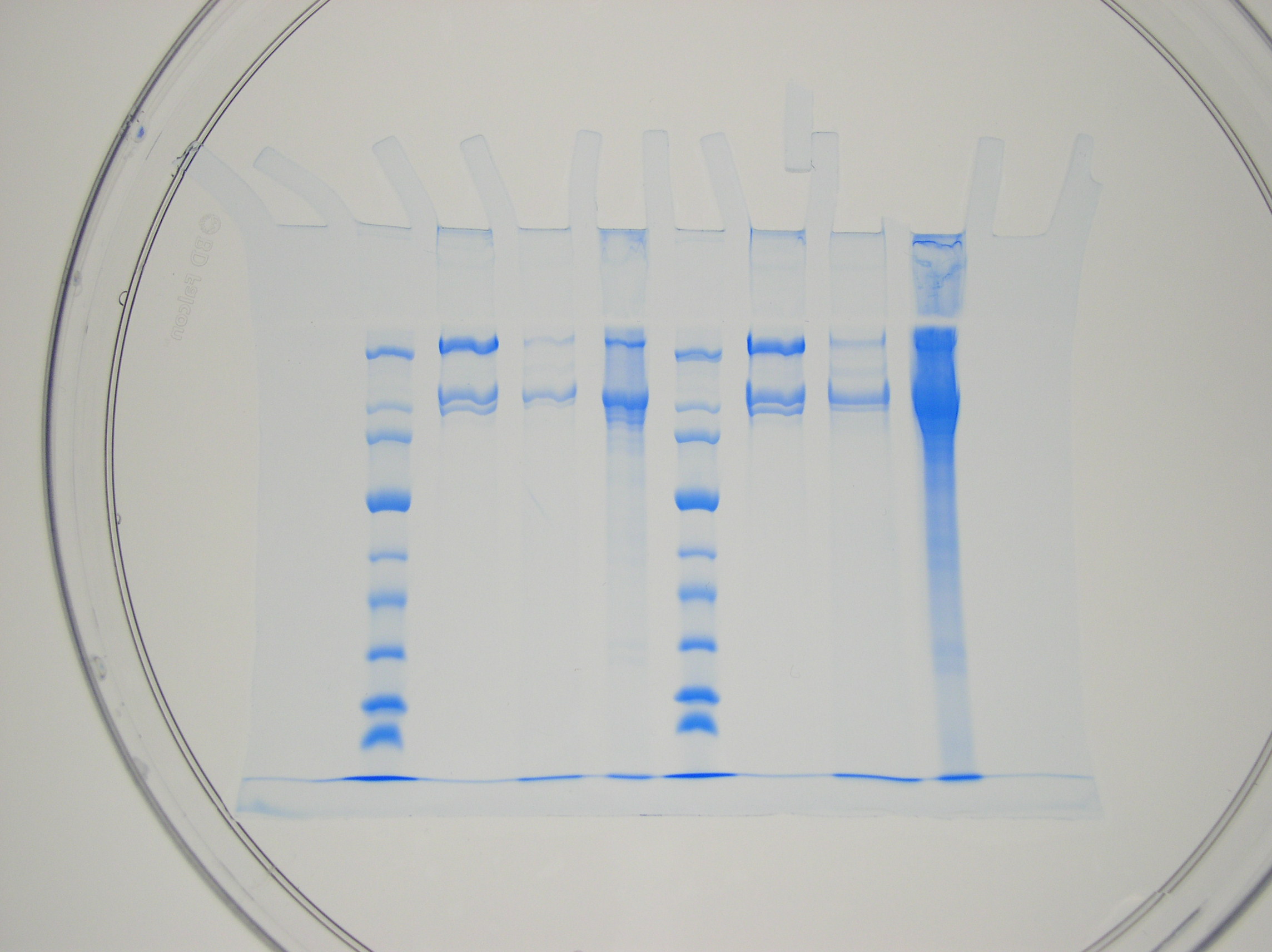

Supplement: S2 Fig — From left: Lane 1 = molecular marker, Lane 2 = type I collagen, Lane 3 = type I collagen, Lane 4 = collagen type III, Lane 5 = marker, Lane 6 = collagen type I (rat), Lane 7 = collagen type I (human). Lanes 4 and 5 are used in S1 Fig. (JPG) [file pone.0250544.s003.JPG]

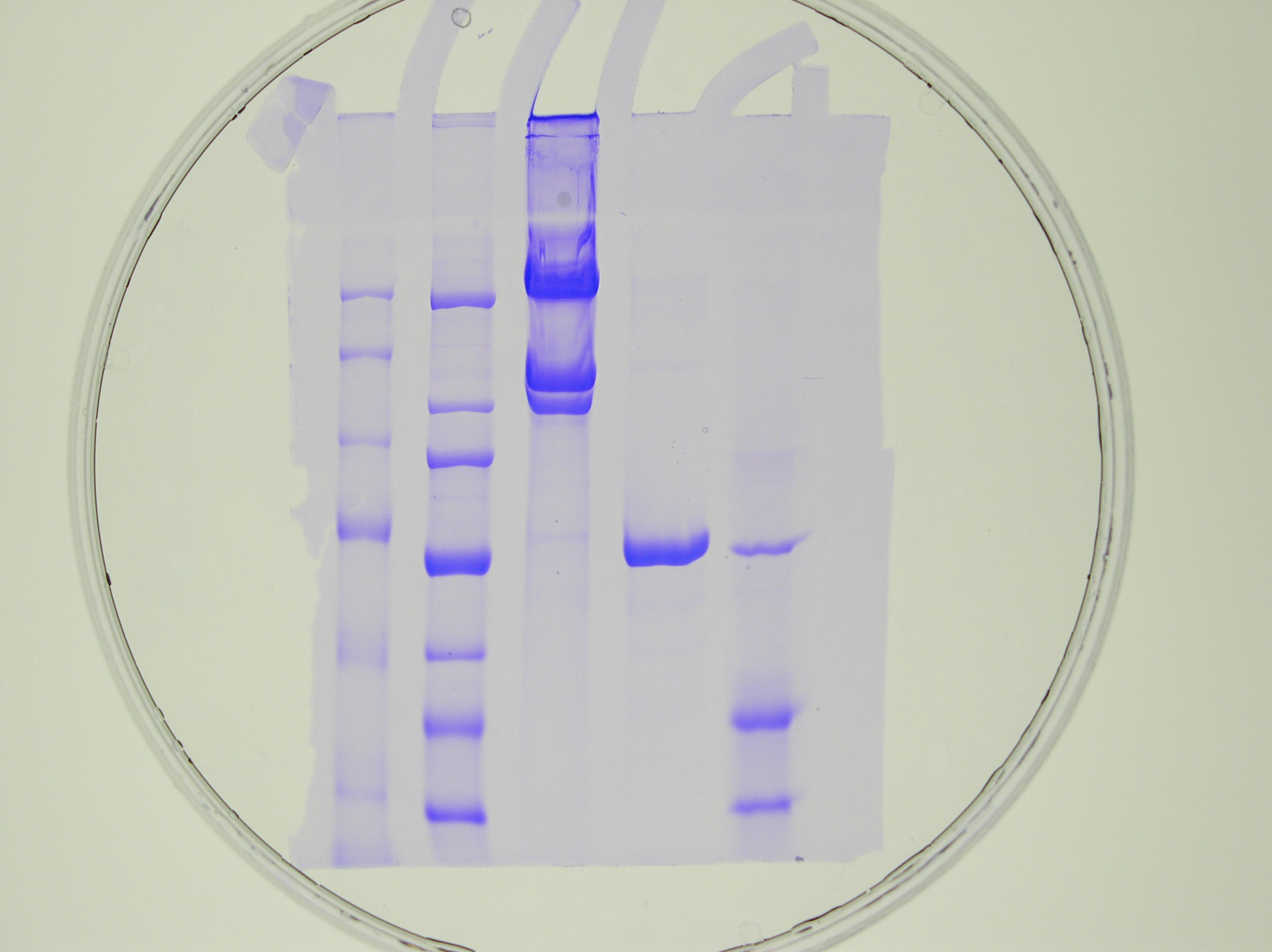

Supplement: S3 Fig — From left: Lane 1 = high range molecular marker, Lane 2 = molecular marker, Lane 3 = collagen type III, Lane 4 = BSA, Lane 5 = low range molecular marker. The rest lanes of the gel were empty. Lanes 2 and 3 are used in S1 Fig. (JPG) [file pone.0250544.s004.JPG]
